# The membrane-actin linker ezrin acts as a sliding anchor

**DOI:** 10.1126/sciadv.abo2779

**Published:** 2022-08-05

**Authors:** Elgin Korkmazhan, Alexander R. Dunn

**Affiliations:** ^1^ Department of Chemical Engineering, Stanford University, Stanford, CA 94305 USA.; ^2^ Graduate Program in Biophysics, Stanford University, Stanford, CA 94305 USA.

## Abstract

Protein linkages to filamentous (F)–actin provide the cell membrane with mechanical stability and support intricate membrane architectures. However, the actin cytoskeleton is highly dynamic and undergoes rapid changes in shape during cell motility and other processes. The molecular mechanisms that generate a mechanically robust yet fluid connection between the membrane and actin cytoskeleton remain poorly understood. Here, we adapted a single-molecule optical trap assay to examine how the prototypical membrane-actin linker ezrin acts to anchor F-actin to the cell membrane. We find that ezrin forms a complex that slides along F-actin over micrometer distances while resisting detachment by forces oriented perpendicular to the filament axis. The ubiquity of ezrin and analogous proteins suggests that sliding anchors such as ezrin may constitute an important but overlooked element in the construction of the actin cytoskeleton.

## Teaser

Ezrin slides along actin filaments to provide a dynamic and robust linkage between the cell membrane and actin cytoskeleton.

## INTRODUCTION

Protein linkages between the cell membrane and the actin cytoskeleton provide the cell membrane with mechanical stability (*1*–*3*) and give rise to intricate membrane architectures such as microvilli in the intestinal epithelia (*3*–*6*). However, the actin cytoskeleton is also dynamic on the seconds time scale, a property that underlies its ability to drive membrane shape changes (*1*) during phagocytosis (*7*, *8*) and amoeboid motility (*9*). Even apparently static structures such as microvilli are maintained via constant F-actin treadmilling (*10*). At present, it is unclear how a mechanically robust yet fluid connection between the membrane and actin cytoskeleton is maintained in these diverse circumstances (*3*, *11*).

A general assumption is that numerous but weak transient cross-links between filamentous actin (F-actin) and the membrane are responsible for this phenomenon (*3*, *12*, *13*). An unexamined, alternative possibility is that proteins linking the membrane and actin cytoskeleton might respond differently to forces oriented parallel versus perpendicular to the membrane plane, potentially allowing F-actin to slide relative to the membrane while maintaining a mechanically stable attachment. Neither of these possibilities has been subject to a direct experimental test. More broadly, to our knowledge, no study to date has systematically examined the response of F-actin–binding proteins to load oriented parallel versus perpendicular to the F-actin filament, a distinction that might be expected to be critical in the case of cytoskeletal membrane anchors.

**Fig. 1.**
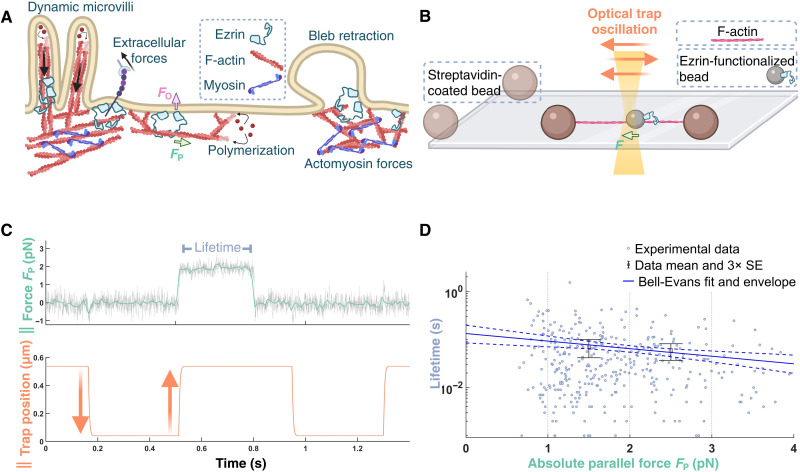
Tightrope assay reveals the force-dependent interaction between F-actin and single ezrin-T567D molecules. (**A**) Ezrin links the cell membrane to the actin cortex, anchors the bases of microvilli, and aids in membrane bleb retraction. In each case, ezrin must resist force orthogonal to the membrane (*F*_O_) while allowing F-actin flow in response to force parallel to the membrane surface (*F*_P_). (**B**) Cartoon of the tightrope assay (not to scale). Single actin filaments are tautly suspended between surface-attached beads. A bead functionalized with ezrin-T567D (here, a single molecule) is captured with the optical trap and oscillated along or perpendicular to the filament. A binding event forces the bead out of the trap center, after which the oscillation is immediately paused to measure the bond lifetime at the given force. The oscillation is resumed following unbinding. (**C**) Example force trace at 100 Hz (green) and 4 kHz (gray) for binding of a single ezrin-T567D molecule to F-actin under load parallel (∥) to the filament axis. (**D**) Bond lifetimes upon parallel loading of single ezrin-T567D molecules (352 events from eight beads from eight flow cells). Mean and error bars (three SEMs) for 1 to 2 pN and 2 to 3 pN are plotted. The data are fit by a Bell-Evans slip bond model (Materials and Methods), where the unbinding rate constant *r* is force dependent as follows $r(F)=r(0)\;e^{\frac{F\;d}{k\;T}}$ (*F*, force; *T*, temperature; *k*, Boltzmann constant; *d*, distance parameter) with a predicted mean lifetime at zero load of 0.13 ± 0.03 s and *d* of 1.5 ± 0.4 nm (errors are SDs). The 95% confidence envelope for the fit (blue dashed lines) and SD for each fit parameter were generated through resampling (Methods).

In this study, we focused on ezrin as a prototypical membrane to F-actin cross-linker. The ezrin-radixin-moesin (ERM) protein family (*4*, *14*) emerged before the divergence of choanoflagellates and metazoans, and its members are present in all sequenced animals, likely acting redundantly in certain contexts (*15*). ERMs link the cell membrane to the actin cortex (Fig. 1A), a thin meshwork of F-actin and myosin II that gives the cell membrane mechanical stability (*1*, *4*, *12*). In addition, ERMs stabilize membrane protrusions such as the microvilli, e.g., in the gut (*6*, *10*, *16*–*18*) and retina (*19*), and play key roles in regulating cell shape change (*20*–*23*) and signal transduction (*4*, *5*, *17*, *24*–*28*). All three ERM proteins are likely to experience mechanical load as part of their physiological functions. For example, ezrin helps drive compaction in the early mammalian embryo (*6*, *29*–*31*), reinforces membrane to F-actin attachment during bleb retraction (*2*), and localizes to microvilli where membrane-actin forces are anticipated to be high (*10*). Consistent with these functions, ezrin knockout mice are born at submendelian ratios and do not survive past weaning due to a failure to form functionally adequate intestinal microvilli despite still having radixin and moesin (*6*). How ezrin maintains a mechanically stable yet dynamic linkage to F-actin is, to our knowledge, not understood.

## RESULTS

As with other ERM proteins, ezrin consists of an N-terminal FERM (4.1 protein, ezrin, radixin, and moesin) domain, which is connected to a C-terminal F-actin–binding region through an α-helical linker region (*4*, *32*, *33*). Ezrin attaches to the membrane through its FERM domain by binding to the membrane lipid phosphatidylinositol 4,5-bisphosphate [PI(4,5)P_2_] and/or to protein partners. Its F-actin–binding activity can be masked in an autoinhibited state, where its F-actin–binding region interacts with the FERM domain (*4*, *33*, *34*). PI(4,5)P_2_ binding and phosphorylation of a conserved threonine residue (T567 in human ezrin) are believed to bias ezrin toward an activated state by helping free its F-actin–binding region (*4*, *33*, *35*–*37*). The phosphomimetic mutant (ezrin-T567D) has been used extensively to study activated ezrin in vivo, and in vitro in the presence of PI(4,5)P_2_ (*35*, *36*). We thus used human ezrin-T567D in the presence of a PI(4,5)P_2_ as a model with which to determine how the interaction of ezrin with F-actin responded to the magnitude and orientation of applied mechanical load.

We adapted a single-molecule optical trap assay (*38*), hereafter termed the “tightrope” assay (*39*), that allows us to apply force to ezrin-T567D bound to F-actin both parallel and perpendicular to the filament axis (Fig. 1B and Materials and Methods). In this assay, streptavidin-coated, 3-μm-diameter beads are adhered to the surface of the coverslip. Biotinylated fluorescent actin filaments are tautly suspended between pairs of beads via the flow generated by rapidly pipetting the F-actin solution through the flow cell. After excess F-actin is washed out, 1-μm beads sparsely functionalized with ezrin-T567D fused to a HaloTag domain (6xHis-HaloTag-ezrin-T567D) are added to the flow cell, along with ~2 μM PI(4,5)P_2_ analog (Materials and Methods). An ezrin-functionalized bead is captured in an optical trap and is then oscillated along or perpendicular to an actin filament. A binding event results in force that pulls the bead out of the optical trap center, which we measure with ~1-ms and ~0.1-pN precisions (Fig. 1C). Upon detection of a binding event, the trap oscillation is temporarily stopped, and the lifetime of the bond at the bound force is recorded (Materials and Methods).

We first examined the effect of parallel load on the interaction of single molecules of ezrin-T567D with F-actin. To ensure that a given optically trapped bead that showed binding activity most likely contained only one active molecule, we labeled beads with low concentrations of ezrin-T567D, such that ~90% of beads showed no binding activity (Materials and Methods). Consistent with Poisson statistics (Materials and Methods and table S1), experiments at this labeling ratio revealed that ~8% of all tested beads exhibited either solely single-step unbinding events or a mixture of single- and double-step unbinding events, as expected from beads containing one and two ezrin-T567D molecules, respectively, where each molecule had a single actin-binding site (Fig. 1C). We thus interpreted the events from beads exhibiting solely single-step unbinding to be from a single ezrin-T567D molecule interacting with the actin filament. Measurements from these beads revealed a particularly weak interaction, with a <100-ms mean binding lifetime when bearing 0.5 to 4 pN, forces comparable to those generated by individual myosin motor domains (Fig. 1D) (*40*–*42*). These lifetimes are consistent with atomic force microscopy experiments that inferred the mean actin-binding lifetime at zero load to be <1 s (*13*).

**Fig. 2.**
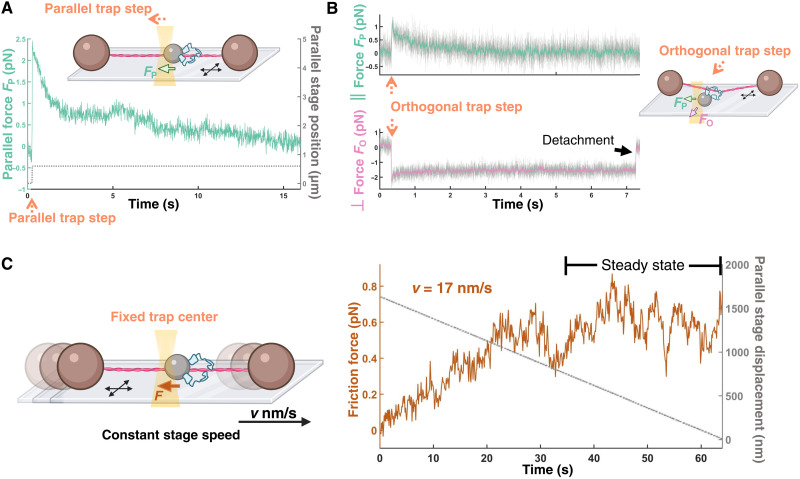
Multiple ezrin-T567D molecules form a sliding anchor on F-actin. (**A**) Force relaxation from ~2 to 0.1 pN following a step in parallel (∥) load and (**B**) from ~1 to 0.1 pN in the presence of a simultaneous orthogonal (⊥) load. Complexes slide along F-actin before unbinding, seen as a step in the orthogonal force trace. Parallel and perpendicular force traces are at 100 Hz (green and magenta, respectively) and 4 kHz [gray; shown only in (B)]. (**C**) Ezrin-T567D complexes slide for multiple micrometers along F-actin. Left: The optical trap is held in a fixed position, while the microscope stage moves at a fixed velocity. Right: This results in a frictional force on the ezrin-T567D complex that asymptotes to a steady state. Force trace is at 10 Hz.

In vivo, ezrin is found as oligomers and more generally in clusters, in addition to monomeric forms (*36*, *43*, *44*). Thus, we next tested the binding of multiple ezrin-T567D molecules to F-actin. We increased the labeling ratio of beads such that ~34% of beads showed F-actin–binding activity (Materials and Methods). According to Poisson statistics, at this labeling ratio, ~27% of beads are expected to be labeled with a single ezrin-T567D molecule, ~6% are expected to contain two molecules, and ~1% more than two. Consistent with this expectation, we mostly observed single-step and double-step unbinding for active beads, as expected from beads binding to F-actin with one or two molecules at a time (table S1 and Materials and Methods). However, 4.5% of beads (18 of all 404 beads tested) showed a qualitatively different behavior, in which a step in applied load relaxed back to 0 to 0.1 pN, while the complex was still bound, as opposed to exhibiting step-unbinding (Fig. 2A). This behavior occurred repeatedly for a given bead.

The statistical rarity of beads exhibiting this force relaxation behavior suggested that it arose from multiple ezrin-T567D molecules acting in concert (Materials and Methods). These complexes could still relax force parallel to the filament axis to ~0 pN even in the presence of perpendicular load (Fig. 2B). This observation suggested the ability of the complexes to slide along the filament without transient detachment events that would otherwise result in irreversible detachment in the presence of perpendicular load. Consistently, we found that these complexes remained stably associated with F-actin for tens of seconds and over micrometer distances when the stage was translated at constant velocity and that they could repeat this behavior for multiple such ramps in succession (Fig. 2C and fig. S1) before exhibiting step unbinding from the filament.

**Fig. 3.**
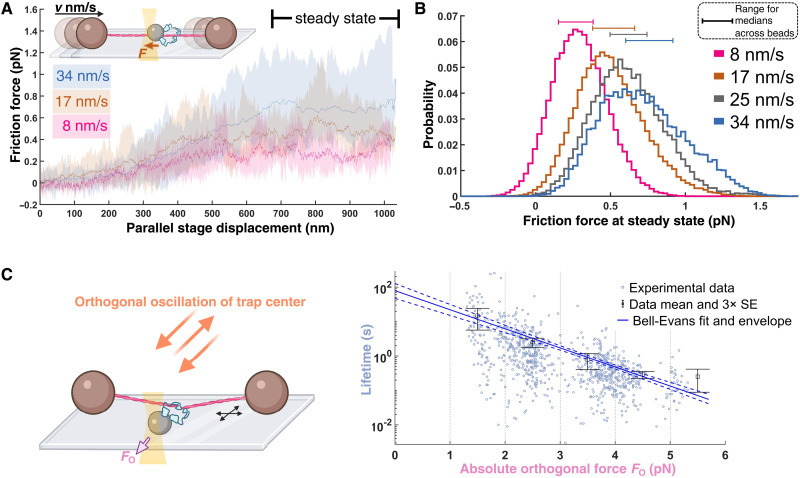
Characterization of the minimal sliding complex. (**A**) Constant stage velocity experiment. Inset: The stage is moved at a triangular wave pattern at a set speed. Example data: Average forces for a given phase of a stage displacement for a representative sliding complex (average across 4, 11, and 14 ramping periods at 8, 17, and 34 nm/s, respectively). Envelopes are bounded by maximum and minimum values. Data were mean-filtered (200-ms window size) before processing for this panel. (**B**) Pooled steady-state friction force distribution (nine beads from nine flow cells; boxcar-averaged at 100 Hz; see Materials and Methods) for the minimal sliding complex. The horizontal line of the same color above each curve shows the range (minimum to maximum) of median friction forces across beads for the corresponding velocity. A two-sample Kolmogorov-Smirnov test is applied to compare each pair of average distributions of the different speeds, generating a *P* value of <0.03 across all pairs. (**C**) Lifetimes under perpendicular loading of the minimal sliding complex (870 events from five beads from five flow cells), with mean and error bars (three SEs of mean) for each 1 pN interval. The data are fit by a Bell-Evans slip bond model (Materials and Methods), where the unbinding rate constant *r* is force dependent as follows $r(F)=r(0)\;e^{\frac{F\;d}{k\;T}}$ (*F*, force; *T*, temperature; *k*, Boltzmann constant; *d*, distance parameter) with a predicted mean lifetime at zero load of 79 ± 21 s and *d* of 5.2 ± 0.3 nm (errors are SDs). The 95% confidence envelope for the fit (blue dashed lines) and SD for each fit parameter were generated through resampling (Materials and Methods).

To determine the characteristics of the minimal ezrin assembly that would support sliding, we next performed experiments at limiting ezrin-T567D labeling concentrations, such that sliding was rare (6 of 289 beads examined). Of these, five beads, from independent bead preparations, produced matching steady-state friction forces for a given velocity (Fig. 3A, Materials and Methods, and fig. S2) and exhibited step unbinding. Both observations are consistent with the presence of a defined, minimal ezrin-T567D cluster with set composition and behavior. Similar steady-state friction values and single-step detachment were likewise observed for a subset of beads labeled with higher ezrin-T567D concentrations (60% of all sliding beads and 2% of all beads). Data pooled from these complexes yielded narrowly distributed steady-state friction values that increased monotonically with increasing velocity, as would be expected from a well-defined complex (Fig. 3B). The binding lifetimes for the minimal sliding complex placed under orthogonal load were well described as a slip bond with an extrapolated mean lifetime at zero load of ~80 s (Fig. 3C), again consistent with the presence of an underlying homogeneous population. In summary, these observations support the presence of a minimal complex that is homogenous in nature and able to slide along F-actin for tens of seconds without unbinding.

**Fig. 4.**
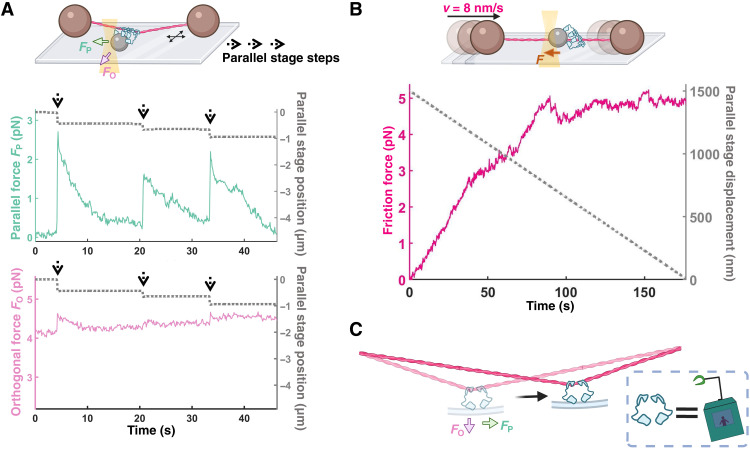
Stronger sliding anchors form when multiple ezrin-T567D molecules work together. (**A**) Parallel force relaxation (green) by a nonminimal sliding complex under repeated step loading events (gray) in the presence of a large, constant orthogonal load (~4 pN; magenta). (**B**) Parallel friction force (stage velocity = 8 nm/s) for a nonminimal sliding complex at steady state is an order of magnitude larger than that of the minimal sliding complex at the same velocity. All force traces are at 10 Hz. (**C**) Ezrin forms a minimal sliding complex that slides along an actin filament while resisting detachment, similar to a cable car ziplining on a wire.

It is likely that ensembles of ezrin-T567D molecules larger than the minimal sliding complex work together in vivo. As the bead labeling ratio was increased, we additionally observed sliding assemblies that differed from minimal sliding complexes. These nonminimal sliding assemblies exhibited ~2-fold or greater friction forces (Fig. 4B, Materials and Methods, and fig. S2), increased heterogeneity in their friction force for a given bead (fig. S3), and were able to bear ~4-pN forces in the orthogonal direction for multiple seconds without detaching (Fig. 4A). During constant stage velocity experiments, these “nonminimal” assemblies sometimes exhibited partial, step-unbinding and rebinding events, after which they continued sliding (figs. S3 and S4). Thus, a larger collection of ezrin-T567D molecules than in the minimal sliding complex yielded a sliding connection to F-actin that persisted for multiple minutes and was stable to substantial perpendicular loads.

The ability of the minimal sliding complex to traverse micrometer distances indicates that it does not follow the F-actin helical pitch, which would require the optically trapped bead to spiral around the actin filament tens of times over this distance. Perhaps relatedly, we noted that the relaxation following a step in parallel force was not smooth but exhibited bursts corresponding to bead movements of 10 to 30 nm (figs. S5 and S6 and Materials and Methods). These bursts and pauses potentially reflect the switching of ezrin-T567D contacts from one face of the actin filament to the other, which, if present, would allow the minimal sliding complex to remain continuously attached while sliding over micron distances.

## DISCUSSION

Previous studies suggest that ERMs play a crucial role in mechanically integrating the actin cytoskeleton to the cell membrane while simultaneously allowing the rapid remodeling of both (Fig. 1A) (*1*, *2*, *4*). Here, we find that a minimal complex composed of multiple ezrins can slide continuously along actin filaments for tens of seconds and multiple micrometers while sustaining physiologically relevant perpendicular loads (Fig. 4C). This functionality explains how ezrin can mediate stable attachment of the cell membrane and cytoskeleton while allowing the two to slide relative to each other, for example during bleb retraction (*2*, *45*) and compaction in the mouse blastocyst (*31*) (Fig. 1A). Ezrin’s ability to act as a sliding anchor is likewise consistent with its prominent role in microvilli, where it provides stable membrane attachment despite constant treadmilling movement of F-actin toward the microvillus base (Fig. 1A) (*10*).

While we do not know the exact composition of the minimal sliding complex, based on the statistics of our bead labeling, we speculate that it may consist of two ezrin molecules. The two molecules may potentially cooperate to slide when in sufficiently close proximity by simple multivalent unbinding and rebinding. However, it is also possible that sliding reflects the activity of a conformationally distinct complex with kinetic properties distinct from the ezrin monomers. Future studies are needed to determine the nature of the minimal sliding complex, and assuming that it corresponds to a defined assembly, whether it shares the antiparallel organization proposed for ezrin dimers in different contexts (*32*, *36*). Emerging single-particle cryo–electron microscopy approaches to studying actin-binding proteins may be particularly useful in this regard (*46*). How and whether pretension on the actin filament may affect ezrin’s F-actin–binding properties likewise provides an interesting direction for future investigations.

Although the biochemical nature of the minimal sliding complex remains unclear, available evidence strongly implies the functional importance of ezrin oligomerization in vivo. Quantitative fluorescence recovery after photobleaching (FRAP) experiments in epithelial cell microvilli suggest the presence of an additional kinetic step beyond T567 phosphorylation before full ezrin activation. This step could potentially correspond to the recruitment and arrangement of multiple ezrin molecules to enable sliding, as suggested by our results (*47*). Of note, ezrin appears to form clusters of seemingly arbitrary size on membranes in vitro (*36*, *48*, *49*), ezrin interaction partners such as focal adhesion kinase (FAK) and l-selectin cluster in vivo (*25*, *28*), and ezrin has been proposed to facilitate FAK activation through promoting clustering of FAK (*28*). It seems likely that PI(4,5)P_2_-rich membrane domains may regulate ezrin cluster formation as well (*50*). Last, a recent study directly imaged ezrin itself during polarization of the mouse embryo at the eight-cell stage, where it strikingly formed growing clusters at the apical membrane (*44*). Elucidating the nanoscale organization of ezrin in its cellular context will be important in understanding how it and related ERM proteins fulfill their diverse cellular functions.

To our knowledge, our study constitutes the first systematic examination of the response of an F-actin–binding protein to loading at varying angles relative to the actin filament axis. Given these results, it seems plausible that other proteins that slide along polynucleotide and cytoskeletal tracks may exhibit unexpected, angle-dependent responses to mechanical loads (*51*–*58*). In particular, whether other cortical anchors may exhibit variations on the sliding anchoring behavior we characterize here represents an attractive target for future work. It will likewise be interesting to investigate the molecular mechanisms by which ezrin and analogous proteins (*3*, *4*, *59*) interact with force-generating membrane anchors, for example, myosin I, to generate the diverse repertoire of cell shapes and dynamics that characterize animal cells (*1*, *2*, *60*–*64*). More broadly, our and other studies (*60*, *62*, *65*) suggest that the emergent properties of the cell cortex may reflect mechanical anisotropies built in at the level of its individual molecular components, only some of which are currently known.

## MATERIALS AND METHODS

### Protein expression construct

The following in-frame fusion protein construct was expressed using a pET28a vector: MGSS-6xHis-8xGS-HaloTag-2xG-TEVsite–GGGSGGGGSGGGGSGG-ezrin-T567D, where TEV site is the TEV recognition and the cleavage site DYDIPTTENLYFQG. The human ezrin-T567D sequence used was MPKPINVRVTTMDAELEFAIQPNTTGKQLFDQVVKTIGLREVWYFGLHYVDNKGFPTWLKLDKKVSAQEVRKENPLQFKFRAKFYPEDVAEELIQDITQKLFFLQVKEGILSDEIYCPPETAVLLGSYAVQAKFGDYNKEVHKSGYLSSERLIPQRVMDQHKLTRDQWEDRIQVWHAEHRGMLKDNAMLEYLKIAQDLEMYGINYFEIKNKKGTDLWLGVDALGLNIYEKDDKLTPKIGFPWSEIRNISFNDKKFVIKPIDKKAPDFVFYAPRLRINKRILQLCMGNHELYMRRRKPDTIEVQQMKAQAREEKHQKQLERQQLETEKKRRETVEREKEQMMREKEELMLRLQDYEEKTKKAERELSEQIQRALQLEEERKRAQEEAERLEADRMAALRAKEELERQAVDQIKSQEQLAAELAEYTAKIALLEEARRRKEDEVEEWQHRAKEAQDDLVKTKEELHLVMTAPPPPPPPVYEPVSYHVQESLQDEGAEPTGYSAELSSEGIRDDRNEEKRITEAEKNERVQRQLLTLSSELSQARDENKRTHNDIIHNENMRQGRDKYKDLRQIRQGNTKQRIDEFEAL. Cloning and sequence verification were done by Epoch Life Science.

### Protein expression and purification

BL21(DE3) competent bacterial cells were transformed with the ezrin-T567D expression plasmid through heat shock at 42°C, grown in LB broth, and plated on LB agar plates with kanamycin (50 μg/ml) to produce colonies. A single colony was used to grow a culture in LB broth with kanamycin (50 μg/ml) at 37°C and 220 to 240 rpm, and a bacterial stock was made in 25% glycerol and stored at −80°C. For protein production, a starter culture of 5 ml of LB broth with kanamycin (50 μg/ml) was induced with a colony from a plate freshly streaked with the stock and was grown overnight at 37°C in a shaker at 220 to 240 rpm. The starter culture was then used to induce 1 liter of LB broth with kanamycin (50 μg/ml) and grown at 37°C and 220 to 240 rpm. Induction of protein expression was done at an optical density of 0.6 to 0.7 with 0.10 to 0.15 mM isopropyl-β-d-thiogalactopyranoside after the culture was kept at 4°C for 5 min. The culture was then moved to 18°C, where it was kept on a shaker at 220 to 240 rpm for ~16 hours.

Cells were then collected by centrifugation at 6000*g* for 20 min at 4°C, and the centrifuged cell pellets were kept at −80°C for a week. For lysis, the centrifuge bottles were moved to ice, and the supernatant medium was discarded. While on ice, the pellets were resuspended by adding, per pellet of 500 ml of culture, a total 10 ml of lysis buffer (300 mM NaCl, 50 mM NaH_2_PO_4_, and 10 mM imidazole, brought to pH 8 using NaOH) with phenylmethylsulfonyl fluoride (14 μg/ml; 50-103-5662, Thermo Fisher Scientific), lysozyme type VI (0.2 mg/ml; 76177-422, VWR), deoxyribonuclease I (10 mU/μl; 4536282001, MilliporeSigma), and 1 cOmplete EDTA-free protease inhibitor cocktail tablet (11873580001, MilliporeSigma).

The suspension was moved to a 360 rotary shaker for 30 to 45 min at 4°C, after which a 130-W ultrasonic processor (VCX 130, Sonics) was used to further lyse the cells on ice in a 4°C cold room. Per cycle of sonication, we used a 30% amplitude, pulsed as 1-s off and 1-s on for a total of 30 s of sonication. A total of ~10 cycles were done in total and in between cycles; we ensured that the temperature remained below ~10°C using an infrared thermometer.

Following sonication, the lysate was centrifuged at 12,000*g* for 30 min at 4°C in a 50-ml Falcon tube. The supernatant was collected, kept on ice, sterile-filtered, and then incubated with 2 ml of HisPur Ni-NTA resin (88221, Thermo Fisher Scientific) slurry per supernatant from 500 ml of culture for 1.30 to 2 hours at 4°C on a 360 rotary shaker.

A gravity flow column with 5-ml capacity (29922, Thermo Fisher Scientific) was assembled in a 4°C cold room, and the bead suspension was loaded onto the column, allowing the solution to run through without letting the beads dry. Following loading, the column was washed with 7 ml of wash A buffer [300 mM NaCl, 50 mM NaH_2_PO_4_, and 20 mM imidazole (pH 7.4), with 0.7 mM freshly added β-mercaptoethanol], 7 ml of wash B buffer [phosphate-buffered saline (PBS), 1 M NaCl, and 0.002 to 0.005% Tween, sterile-filtered), and, lastly, 7 ml of wash A buffer. This was followed by elution with a total of 8 to 9 ml of elution buffer [300 mM NaCl, 50 mM NaH_2_PO_4_, and 250 mM imidazole (pH 7.4), with 0.7 mM freshly added β-mercaptoethanol]. In preparation for ion exchange chromatography (IEC), the eluate from the Ni-NTA resin was pooled and buffer-exchanged into IEC buffer 1 (20 mM tris, pH 8, sterile-filtered) via two concentration and dilution steps at 4°C using Amicon Ultra 2-ml 10-kDa centrifugal filters (MilliporeSigma), which we estimate to have resulted in a ~3-fold dilution in the salt concentration. The protein solution was sterile-filtered, loaded on an anion exchange column (Mono Q 5/50 GL, GE Healthcare) in IEC buffer 1, and subsequently eluted with a linear gradient of IEC buffer 2 [20 mM tris and 0.98 M NaCl (pH 8), sterile-filtered]. Following SDS–polyacrylamide gel electrophoresis (SDS-PAGE) analysis, two consecutive 300-μl fractions eluting at ~220 mM NaCl were found to be enriched for the desired construct, with minimal contaminants of differing molecular weights. These fractions were pooled and loaded onto a Superdex 200 Increase 10/300 GL column (GE Healthcare) equilibrated with protein storage buffer [20 mM tris, 150 mM NaCl (pH 8), and 1 mM fresh dithiothreitol (DTT), sterile-filtered] for size exclusion chromatography. Consecutive fractions corresponding to protein of the anticipated molecular weight were pooled and saved. The pooled protein solution (~600 nM) was stored at 4°C overnight, aliquoted, and snap-frozen into −80°C the following day. Tris buffers used in protein purification were brought to the desired pH either by mixing equimolar solutions of tris base (BP154-1, Fisher) with tris hydrochloride (BP153-1, Fisher) or through titration with hydrochloric acid. IEC and SEC were conducted using the GE AKTA PURE Fast Protein Liquid Chromatography System in a 4°C cold room at the Stanford ChEM-H Macromolecular Structure Knowledge Center.

### Optical trap setup

We performed our optical trap experiments on a commercial Lumicks C-Trap, which uses a 10-W infrared (1064 nm) laser focused through a Nikon 60× objective [CFI Plan Apo, numerical aperture (NA) of 1.2] to produce two traps with one (trap 1) more sensitive than the other (trap 2). An epifluorescence imaging setup was added to image fluorescent actin filaments using a 532-nm laser (Coherent OBIS 532-80-LS) and a scientific complementary metal-oxide semiconductor camera (PCO).

### Buffer components

The source of following chemicals was Fisher Scientific: MgCl_2_ (600-30-96), CaCl_2_ (C79-500), KCl (P217-500), and tris base (BP154-1). Tris buffer stocks were brought to the desired pH by titration with hydrochloric acid (except for protein purification; see above). F-buffer was prepared as 20 mM tris, 50 mM KCl, 2 mM MgCl_2_, and 0.2 mM CaCl_2_ (pH 8), sterile-filtered, and either kept at 4°C or stored aliquoted at −80°C. Ten times concentrated F-buffer was prepared using the same reagents, sterile-filtered, and stored aliquoted at −80°C. FBSA buffer consisted of F-buffer with ultrapure bovine serum albumin (BSA; 1 mg/ml; MCLAB, UBSA-100) and either stored at 4°C for ~2 days or stored aliquoted at −80°C. DTT (DTT100, GoldBio) was dissolved in water, sterile-filtered, and stored as 1 M aliquots at −80°C. Two sources of adenosine 5′-triphosphate (ATP) were both stored as aliquots at 100 mM at −80°C. ATP from Calbiochem (1191) was dissolved and adjusted to pH 8 with NaOH. ATP from Thermo Fisher Scientific (R0441) was bought in solution form already adjusted to pH 7.3 to 7.5 with NaOH and stored at −20°C before aliquoting into −80°C.

PI(4,5)P_2_ diC4 (P-4504, Echelon Biosciences) was either dissolved directly in F-buffer and stored at −80°C (as aliquots or as stock) or dissolved in ultrapure water, stored at −80°C, and diluted 20× in F-buffer before usage (see the “Tightrope assay” section for details of usage). Phalloidin (NC1108931, Fisher Scientific) was stored in aliquots at −80°C dissolved to 1 mM in water or F-buffer.

For oxygen scavenging, we used a pyranose oxidase and catalase (POC) system (*66*) plus Trolox. Glucose (anhydrous dextrose; BP350500, Fisher) was dissolved to 60% in F-buffer, sterile-filtered, and stored aliquoted at −80°C. We prepared a stock solution of pyranose oxidase (P4234-250UN, MilliporeSigma), catalase (50 kU/ml; C40-100 mg, MilliporeSigma), and BSA (~0.15 to 0.5 mg/ml; UBSA-100, MCLAB) in F-buffer that was sterile-filtered, aliquoted, snap-frozen, and kept at −80°C. Trolox (648471, MilliporeSigma) was dissolved in F-buffer to 120 mM and stored aliquoted at −80°C.

### Preparation of fluorescent biotinylated F-actin

Lyophilized rhodamine phalloidin (PHDR1; Cytoskeleton) was resuspended to ~800 μM using 8.7 μl of methanol (ACS Spectrophotometric Grade, ≥99.9%; Honeywell Riedel-de Haën), rapidly aliquoted in ~0.5-μl volumes into tubes, and stored in −20°C, to be later mixed with F-actin as below.

Actin was purified from rabbit skeletal muscle, stored, and biotinylated using biotin–*N*-hydroxysuccinimide (NHS; 203118, Sigma-Aldrich) exactly as previously described (*65*). The biotinylated actin was snap-frozen at a concentration of ~1 mg/ml (24 μM) in ~20 μl of aliquots in G-buffer [5 mM tris (pH 8.0), 0.2 mM CaCl_2_, and 0.2 mM ATP] with 1 mM DTT. Before polymerizing biotinylated actin, an aliquot was thawed on ice for ~30 min, and 20 μl from the aliquot was centrifuged in a TLA100.2 rotor at 60,000 rpm for 10 min at 4°C to remove aggregates. The supernatant was moved to a plastic tube, during which the total remaining volume was estimated. F-buffer (101) containing 10 mM DTT and 10 mM ATP was then added at a volume of one-ninth that of the supernatant, inducing polymerization at the actin concentration of ~22 μM. This was mixed and polymerized while on a rotator at room temperature for ~40 min, after which it was diluted to 110 μl (~3.5 μM) using F-buffer with 1 mM DTT and 1 mM ATP and transferred to a tube of a rhodamine phalloidin aliquot containing 0.5 μl of ~800 μM rhodamine phalloidin. This fluorescent biotinylated F-actin stock was kept on ice at 4°C for 1 to 2 days for rhodamine phalloidin to incorporate into filaments, after which it was kept on ice at 4°C and used in the tightrope optical trap assay within ~2 to 3 weeks.

### Tightrope assay

#### Functionalization of trapping beads

All centrifugations were done at 3000*g* for 5 min on a benchtop centrifuge, and all sonication steps were performed with a bath sonicator. When removing supernatants from bead pellets, a minimal amount of solution was left to keep the beads wet.

BSA (UBSA-100, MCLAB) was functionalized with Halo-ligand [HaloTag Succinimidyl Ester (O4) Ligand; P6751, Promega]. Fresh Halo-ligand was thawed to room temperature before opening and dissolved to 80 mM in anhydrous dimethyl sulfoxide (DMSO; 900645, MilliporeSigma) from a freshly opened ampule. This was then mixed with a 100 μM BSA solution in PBS (pH 7.4) to achieve 3 mM Halo-ligand at less than 4% DMSO per reaction tube. The experimental reactions were paired with control reactions in parallel where the DMSO contained no Halo-ligand. The reaction mixture tubes were incubated for 2 hours 30 min at room temperature on a shaker and 3 hours 30 min at 4°C on a 360° rotator. During the incubation at 4°C, samples from each of the experimental and control reaction mixtures were buffer-exchanged into PBS (PD Minitrap G-25; GE28-9180-07, MilliporeSigma) and subsequently reacted with a HaloTag-fused protein. This showed >1 new molecular species for the experimental mixture in SDS-PAGE analysis, indicative of multiple Halo-ligand links per BSA molecule. Aliquots of the reaction mixture were snap-frozen and stored at −80°C, where the nondesalted aliquots were used in the reactions with beads as described below, referred to as BSA–Halo-ligand for the experimental and BSA-control for the control solution aliquots. The buffer-exchanged aliquots were used in SDS-PAGE analysis to recheck the high labeling efficiency of BSA, to confirm the preservation of its cross-linking activity to HaloTag-fused proteins, and to confirm the functionality of the HaloTag domain in the ezrin construct.

To attach BSA–Halo-ligand to beads used for optical trapping, we first activated carboxyl-functionalized silica beads (mean diameter of 1.0 μm; SC04000, Bangs Laboratories) with EDC [1-ethyl-3-(3-dimethylaminopropyl)carbodiimide hydrochloride; PG82079, Thermo Fisher Scientific] and sulfo-NHS (*N*-hydroxysulfosuccinimide; PG82071, Thermo Fisher Scientific) as follows: Carboxyl silica beads were resuspended at 30 mg/ml in MES buffer [sterile-filtered 0.1 M MES and 0.9% sodium chloride (pH 4.7) made in ultrapure water with BupH MES-Buffered Saline Packs; 28390, Thermo Fisher Scientific], vortexed, and bath-sonicated for 15 min. This batch was then split and diluted to 9 mg/ml in 1 ml of MES buffer per tube. The following wash procedure was done three times per tube: 30-s sonication, centrifugation to pellet the beads, removal of supernatant, and resuspension to 9 mg/ml in MES buffer. After an additional 30-s sonication, sulfo-NHS and then EDC—each freshly and separately dissolved in MES buffer at 190 and 230 mM concentrations, respectively—were sequentially added to the tubes, which had a final concentration of 43 mM Sulfo-NHS, 30 mM EDC, and beads (5.8 mg/ml) in a final volume of 1.55 ml per tube. Each tube was then vortexed, bath-sonicated for 2 min, and kept on a shaker for 15 to 20 min where additional manual mixing of tubes via inversion and vortexing was done during the incubation, with a 30-s bath sonication toward the end. The activated beads were then centrifuged and resuspended in PBS (pH 7.4) after removal of the supernatant. This was repeated once more, after which beads from all tubes were pooled together, sonicated for 2 min, mixed, and then split into tubes for reaction with BSA–Halo-ligand or BSA-control. The final reaction mixture per tube contained beads (9 mg/ml) and 17 μM BSA–halo-ligand or BSA-control in PBS (pH ~7.4), which was sonicated, vortexed, and kept on a high-angle shaker to react for 3 hours at room temperature. Following centrifugation, the supernatant was removed, and bead pellet was resuspended in PBS with 20 to 40 mM glycine (sterile-filtered, pH ~7.2) to quench the reaction, sonicated for 30 s, vortexed, and incubated while mixing for 35 min, with 1 mM DTT added for the last 10 min. The beads were then washed twice with PBS with 1 mM DTT through centrifugation and finally resuspended for passivation in 0.5% casein (from C4765, MilliporeSigma; stored at 4°C), 0.5% BSA, and 1 mM DTT in a final 85% PBS and 15% water mixture at bead concentration (9 mg/ml). The suspensions were vortexed, sonicated for 2 min, and incubated for 2 hours while mixing, with extra vortexing and sonication in the middle of the incubation. The beads were washed twice via centrifugation and finally resuspended at beads (9 mg/ml) in 0.1% BSA, 0.05% casein, and 1 mM DTT in PBS. The bead solution was mixed, sonicated for 2 min, snap-frozen in 40 μl of aliquots, and stored at −80°C.

Following thawing, a second round of passivation was performed as follows. Pluronic F-127 was prepared within 3 days at 5% in F-buffer and sterile-filtered. BSA–Halo-ligand beads and BSA-control beads were thawed, and each was resuspended at beads (1.4 mg/ml) in 1% Pluronic F-127, 0.016% BSA, and 0.017% casein in a mixture of 80% PBS and 20% F-buffer. The mixture was sonicated for 40 s and mixed on a 360° rotator at room temperature for 1 to 1.30 hours. The beads were then centrifuged and exchanged into 2% casein and 0.5% BSA and mixed on a 360 rotator at room temperature for 50 min. Last, the beads were washed with PBS through two centrifugations; resuspended in PBS that was brought to 2.6 mg/ml beads, 0.1% BSA, and 0.08% casein; mixed; sonicated for 50 s; snap-frozen in 50 μl of aliquots; and stored at −80°C (Table 1).

**Table 1.** Summary of passivation and storage steps for functionalized beads.

**Passivation (P) & Storage (S) steps following quenching**	**Passivation agents in solution**		
	**BSA (%)**	**Casein (%)**	**Pluronic F-127 (%)**
P1 (2 hr)	0.5	0.5	0
S1	0.1	0.05	0
P2.1 (1-1.30 hr)	0.016	0.017	1
P2.2 (50 min)	0.5	2	0
S2	0.1	0.08	0

We note that only for batch VII (see table S1 and the “Labeling trapping beads with HaloTag fusion protein” section), the BSA–Halo-ligand beads were prepared differently, with main differences being nonspecific attachment of BSA to silica (noncarboxyl) beads, on-bead functionalization of Halo-ligand to BSA, and passivation of beads solely with BSA. We did not exclude this batch in our tallying as we did not observe any qualitative differences in F-actin–binding behavior (i.e., binding lifetimes and sliding behavior) and thus used it, with the other batches, in deducing the percentage of sliding and stepwise detaching complexes for the given bead activity level (next section).

At the ~90% bead inactivity ratio, for ezrin-functionalized beads showing single-step unbinding events, we detected an average of 2.2 events per minute, with an average of 20-min data collection per bead. When assayed similarly for a comparable length of time, control beads reproducibly produced zero events per data collection, putting a ceiling on the background binding rate that we interpret as negligible. When we functionalized the beads with more ezrin molecules, progressively increasing bead activity ratios up to ~34%, the fraction of beads exhibiting step events increased (table S1), indicating that these events arise from specific, ezrin-dependent events.

#### Labeling trapping beads with HaloTag fusion protein

Here, we describe the general protocol for attaching HaloTag fusion proteins to the BSA–Halo-ligand or BSA-control beads, with specific details per batch given in table S1. In summary, bead batches at different ezrin-T567D labeling ratios were made by combining FBSA (see the “Buffer components” section) with the components for incubation for 2 to 75 min at room temperature in final volume at 50 to 200 μl and the following ranges in final concentrations: 4 to 150 nM ezrin-T567D, beads (0.4 to 2 mg/ml), and 1 mM fresh DTT. At the end of the incubation, the mixture was centrifuged at 3000*g*, 5 min. The bead pellet was washed at room temperature by repeatedly removing the supernatant and flowing in 90 μl of FBSA with 1 mM DTT without disturbing the pellet, for a total of 1.5 ml of FBSA wash. A minimal amount of supernatant at each step was left to keep the beads wet. Last, the washed pellet was resuspended in FBSA with 1 mM DTT to a bead concentration of ~0.2 mg/ml. The batch resuspension was bath-sonicated up to two times for ~15 s each. The resuspended beads were then kept at 4°C for up to ~3 hours, during which they were used for experiments or snap-frozen in 4 to 8 μl of aliquots. Multiple experiments showed no difference in actin-binding behavior between frozen versus nonfrozen beads.

#### Reagent preparation

The stock of 3-μm diameter streptavidin-coated polystyrene beads (CP01005, Bangs Laboratories) was diluted 1:10 in F-buffer for the final working stock (beads of 1 mg/ml), after washing and sonication as follows: Each washing step consisted of centrifugation at 3000*g* for 5 min in a tabletop centrifuge and removal of the supernatant, which was followed by resuspension in fresh solution. The bead stock, kept at 4°C, was first diluted 1:10 in ultrapure water by pipetting 50 μl of well-vortexed beads into 500 μl of ultrapure water in an Eppendorf tube and kept at the same dilution whenever resuspended after washes. The bead suspension was then washed, resuspended in ultrapure water twice, sonicated in an ultrasonic bath for 5 min, rewashed, resuspended in ultrapure water, sonicated for 5 min, and finally washed and resuspended in FB twice. The tube was then sonicated for ~5 min in an ultrasonic bath. This working stock was kept on ice at 4°C for 2 to 3 weeks for use in the tightrope optical trap assay.

Pluronic F-127 used for flow cell passivation was dissolved at 5% (w/v) in F-buffer, kept at 4°C until bubbles were mostly removed and then sterile-filtered, and stored as aliquots at 4°C. For experiments with bead batches I to VI (table S1), 5% casein solution (C4765, MilliporeSigma) was aliquoted, snap-frozen, and stored at −80°C, while it was stored at 4°C without freezing for older experiments.

#### Flow cell protocol

Microscope slides (12-544, Fisherbrand Premium Plain Glass Microscope Slides), microscope coverslips (48366-227, VWR), and double-sided tape (Scotch) were used to form a flow cell that held a volume of ~10 to 15 μl of solution as described previously (*38*). Briefly, two stripes of tape of length at ~30 mm were laid parallel on the long axis of the slide to create a channel in between them of width at ~5 mm. A coverslip was then placed on top of the tape, and a good contact with the tape was ensured by pressing on the coverslip tape contacts with the back of a marker. This produced a flow cell that held a volume of ~10 to 15 μl of solution.

At most, several hours before optical trap experiments, aliquots of the following solutions were placed on ice or a metal cooling block immersed in ice and discarded within the indicated number of days: 1 M DTT (1 day), 100 mM ATP (1 day), 5% casein (1 day), FBSA (~1 to 3 days), 120 mM Trolox (1 day), POC (1 to 2 days), 60% glucose (1 to 2 days), Pluronic F-127 (~month), 1 mM phalloidin (~month), and F-buffer. Aliquots that were not discarded within the day were kept at 4°C in between experimental days. PI(4,5)P_2_ diC4 was either used in aliquoted forms that were discarded within 2 days or used from stocks thawed for brief durations before being refrozen (see the “Buffer components” section). At the start of experiments, 1 M DTT was diluted to make a 100 mM working stock in FBSA. F-buffer was used for diluting casein to below 5% when needed.

The trapping bead suspension of 4 to 8 μl was either aliquoted from the freshly labeled batch kept on ice or taken from the snap-frozen aliquots ~5 min before start of the first flow cell wash. This aliquot was kept at room temperature and brought to 13 μM PI(4,5)P_2_ diC4 by mixing with one-fourth of its volume of PI(4,5)P_2_ (0.05 mg/ml). Four microliters of this mixture was later combined with other components of the flow cell (see below) to yield a final PI(4,5)P_2_ diC4 concentration of 2.1 μM during optical trapping. The optical trapping of beads was performed in T-buffer (Table 2), the buffer in the enclosed flow cell [0.84% glucose, 0.8 to 0.9 mM Trolox, 10 μM phalloidin, 1 mM ATP, 1 mM DTT, pyranose oxidase (7.50 units/ml), and catalase in FBSA (1 kU/ml)], which was sequentially formed as described below.

**Table 2.** Components of T-buffer and S-buffer.

**T-buffer**		
**Description**	**Components**	
Buffering agent	Tris, 20 mM, pH 8	**S-buffer**
Ions	KCl, 50 mM	
	MgCl_2_, 2 mM	
	CaCl_2_, 0.2 mM	
Nucleotide for F-actin	ATP, 1 mM	
Reducing agent	DTT, 1 mM	
Passivation agent	Bovine Serum Albumin, 1 mg/ml	
F-actin stabilizing agent	Phalloidin, 10 μM	
Triplet scavenger	Trolox, 0.8-0.9 mM	
Oxygen scavenging system	Pyranose oxidase, 7.50 units/ml	
	Catalase, 1 kU/ml	
	Glucose, 0.84%	
Lipid analog	PI(4,5)P_2_ diC4, 2.1 μM	

In practice, we first prepared W-buffer, which is 1.19× the concentration of T-buffer. W-buffer without POC was kept on ice and used for one to three flow cells. Pyranose oxidase and catalase (see the “Buffer components” section) were added immediately before or during each flow cell preparation. The addition of ~4 μl of bead suspension to the ~21 μl of W-buffer aliquot yielded T-buffer with beads.

S-buffer (10 μM phalloidin, 1 mM ATP, and 1 mM DTT in FBSA) was prepared in amounts to be used for one to three flow cells. Fluorescent, biotinylated F-actin from the ~3.5 μM stock was diluted in an S-buffer aliquot, which was then added to the flow cell at step 5 below. The concentration of F-actin in this solution is estimated to be ~30 to 150 nM, optimized for each batch. The ideal flow cell included dumbbells with long filaments (~5 to 20 μm) every few fields of view, with minimal extra F-actin. Each solution to be added was aliquoted in the amounts to be used and moved to room temperature from ice ~5 min before being flowed in to minimize bubble formation due to temperature changes. The addition of solutions to the flow cell was as follows:

1) F-buffer (>50 μl) was added to wash the flow cell.

2) Well-resuspended working stock of streptavidin bead solution (20 μl) was added and incubated for 8 to 12 min for nonspecific attachment to the surface.

3) The flow cell was washed and passivated (see below).

4) FBSA (~42 μl) was added and incubated for ~2 min for further passivation. Beads were mixed into W-buffer to make T-buffer during this incubation step.

5) S-buffer (~18 μl) with F-actin was rapidly added by tilting the slide and ensuring smooth flow.

6) T-buffer (~20 μl) with beads was rapidly added.

7) Vacuum grease was used to seal ends of the flow cell.

The flow cell was incubated with the coverslip facing down during steps 2 and 3 for attachment of beads to the coverslip.

The three different wash and passivation protocols used in step 3 above were one of the following:

1) A total of 40 μl of F-buffer wash and 20 μl of 5% Pluronic with incubation for ~3 min.

2) A total of 20 μl of F-buffer wash and 20 μl of 5% casein with incubation for 1.30 to 2.30 min followed by 20 μl of F-buffer wash and 20 μl of 5% Pluronic with incubation for 1.30 to 2.30 min.

3) A total of 20 μl of F-buffer wash, 20 μl of 0.8 to 5% casein (diluted to <5% with F-buffer) wash, 40 μl of F-buffer wash, and 20 μl of 5% Pluronic with incubation for 3 min.

During optical trapping (see the “Optical trap setup” section), a tightrope, aligned in the *x* axis (long axis of the flow cell), was found by scanning using bright-field microscopy to monitor streptavidin beads and epifluorescence microscopy to monitor F-actin simultaneously. After a suitable tightrope was identified, a trapping bead free in solution was captured into trap 1 (trapping beads stuck to the surface, if present, were not used). Trap 2 was used to remove other trapping beads from the vicinity of the tightrope and to bring beads to trap 1 to minimize moving trap 1. Data to be used for fine calibration during postprocessing were collected with a trapping bead at or near the trap 1 position where experimental data were acquired (see the “Data processing” section). The fine-calibrated trap 1 stiffness was 0.020 to 0.032 pN/nm in the *x* axis and 0.021 to 0.034 pN/nm in the *y* axis. According to the equipartition theorem and assuming an effective stiffness of 0.03 pN, the bead position will fluctuate in position around a potential minimum approximately as a Gaussian distribution with an SD of $\sqrt{\mathrm{kT}/s}$, corresponding to ~11.7 nm (~0.35 pN in force), where *k* is the Boltzmann constant, *T* is temperature, and *s* is stiffness.

##### Step loading experiments

To assay binding lifetimes when nonsliding complexes were loaded parallel to the filament, the bead was first brought in contact with a filament, as detected by the displacement in *y* force of the trap when pushing against the filament, where the *y* axis is orthogonal to the filament. The bead was then kept pressed against the filament at a ~0.1- to 0.3-pN orthogonal force to ensure contact and oscillated along the *x* axis by moving the trap center in alternating steps in +*x* and −*x*, with pauses in between the steps to check for binding above an absolute force threshold. If binding was detected, then the oscillation was automatically stopped until unbinding lowered the force to below the detection threshold (Fig. 1C). For experiments in Fig. 1D, the step heights were 0.35, 0.4, 0.5, or 0.6 μm. Each step was completed within ~10 ms. The force threshold was set to 0.25 pN with the coarse calibration during each experiment, which, upon fine calibration per collected dataset (see the “Data processing” section), was found to be 0.29 pN on average.

##### Constant stage speed experiments

The stage was moved in a triangular wave in the *x* axis at mean ramp speeds of 8.4, 17, 25, and 34 nm/s (less than nm/s variation), with each ramp being of peak-to-peak amplitudes of 0.75 to 2 μm.

##### Binding lifetimes under orthogonal load for sliding complexes

To assay binding lifetimes, positively identified sliding complexes, for which multiple cycles of steady-state data had been collected, were subjected to load orthogonal to the filament axis, and the trap center was moved in a step oscillation perpendicular to the actin filament axis, with loading step heights of 0.9 to 1.6 μm and loading step completion time of ~5 ms. Binding events were scored as occurring when the force on the bead exceeded a threshold of 0.5 pN, which upon fine calibration corresponded to 0.55 pN on average. The center position of the trap and the trap oscillation amplitude was determined before data collection by manually moving the stage perpendicular to the filament axis such that peak forces were 1 to 4 pN. The bead was asymmetrically positioned such that it would approach the filament orthogonally from one side and barely be flush against it at the end of the oscillation.

The longer the tether in the assaying direction, the more accurate our measurements are, as the applied force vector on the bead will lie progressively closer to the *xy* plane. We expect the effective tether length between the bead and the filament in the orthogonal direction during orthogonal load experiments to be ~1 μm. The trap stiffness is 5 to 10 times lower in the *z* axis than *x* or *y* (estimated by Lumicks); thus, when the bead center is not in the same plane as the filament, we expect a mismatch of up to ~20% between the orthogonal force and total net force on the bead in the force range we assay due to displacements of the bead in the *z* axis (*67*–*69*).

### Data processing and analysis

The MATLAB (RRID: SCR_001622) software tweezercalib 2.1 (*70*) was used for fine stiffness calibration of the optical trap where the dependence of hydrodynamic friction on frequency and on the bead’s proximity to the coverslip surface was taken into account, the position detector was treated as a low-pass filter with one parameter, aliasing was accounted for, and the cross-talk between *x* and *y* axes was eliminated. The absolute height of the trapped bead from the surface (typically ~1 to 3 μm from bottom of the bead) was estimated within ~400 nm using a template precreated in the Lumicks software for a surface streptavidin bead. The bead height from the surface for fine-calibration data collected for two beads was not noted. Both beads were determined to be nonminimal sliding complexes. For analysis of these two beads, we assumed a bead height of 2 μm (typical in our experiments) for the fine calibration, which we expect to introduce an inaccuracy of at most ~10% in our force measurement, which does not affect our interpretations.

The data from trap experiments assaying binding lifetimes (parallel or orthogonal) were boxcar-averaged to 1000 Hz, and binding events were detected as follows, where we consider successful binding when the complex remains bound >15 ms following the completion of loading. First, all possible time points where loading of the complex could happen were found by detecting steps in the trap position through the ischange function in MATLAB. Using this information, we scanned time points that corresponded to 15 ms after the completion of a potential loading. When the force along the relevant axis at this time point exceeded threshold A (0.75 pN for parallel and 1.1 pN for orthogonal loading), the event was considered a successful binding, and the lifetime was taken as the time interval starting from this point (that is, after 15 ms) until before the force decreased below threshold B (0.225 pN for parallel and 0.275 pN for orthogonal loading). The force for the binding event was taken to be the average over the lifetime.

To analyze the steady-state friction force of sliding complexes, the turning points of the stage during the triangular wave were either manually or automatically detected to extract the time points where ramping phases started and ended. However, if sliding complexes unbound/rebound during a ramp, then each section of continuous sliding potentially long enough to reach steady state was manually selected. Likewise, if the experiment was compromised for part of the sliding event, for example, by the presence of a nearby diffusing bead, then only the uncompromised part was selected.

The time point at which steady state was reached was determined as follows: The optical trap force time series were boxcar-averaged to 100 Hz, filtered with a moving mean window size of 200 ms, and the first time point where the force fluctuated to 0.22 pN in the opposing direction to the ramping was taken. An additional 400/*v* s, where *v* is the stage speed in nanometer per second, was added to this time point to account for the bead rotation (bead radius of 500 nm). An additional 15 s was further added to further ensure that steady state had been reached. The force traces corresponding to the resulting steady-state time intervals were analyzed as boxcar-averaged to 100 Hz (without any moving mean filtering).

#### Labeling ratio of bead batches and their associated data

Here, we describe the different batches of trapping beads labeled with HaloTag fusion ezrin-T567D used in our experiments (table S1). As described in the “Flow cell protocol” section, we used three different passivation methods at step 3 and found that the casein-based protocols were best at preventing the sticking of beads to the surface or to streptavidin beads. We do not sample stuck beads; thus, excessive sticking of trap beads is potentially problematic when estimating the percentage of active beads for a batch. Control experiments with unlabeled beads (i.e., BSA–Halo-ligand beads with no HaloTag ezrin-T567D during incubation) showed a ~10% ratio of nonspecific sticking of beads to the surface using a casein-based protocol. Per tallied flow cell, we used this control ratio as a guide and noted the ratio of beads stuck to the surface over the course of the experiment to determine until what time point, for a given flow cell, statistics could be safely tallied.

When testing the labeling statistics of beads, we made use of filaments that were taut enough that we could ensure by pushing the bead against the filament at forces of ~0.1 to 0.3 pN, which any active complexes on the bead would likely encounter the filament during the parallel trap oscillation. We used two types of scans, short (~30 s) and long (~1 min). These durations were determined empirically during optimization. Long scans were able to detect whether a bead was in general active, i.e., whether it contained stepping or sliding molecules. However, because of the faster on-rates of sliding complexes compared to stepping complexes, sliding complexes could be easily detected with ~30-s scans alone. Thus, to speed up bead sampling, sometimes, the short scan procedure was used to detect whether a bead contained a sliding complex or not, where a sliding complex was further confirmed by manual movement of the stage and/or steady-state friction experiments. In some datasets, we started off using long scans but then switched to short scans during course of the experiment. In these cases, data from flow cells were divided into two sections during data processing: the first containing the initial long scans and the second containing the short scans. These are referred to as flow cell sections below.

From the casein-based passivation protocols, 26 flow cell sections were used for tallying, where 22 had a stuck bead ratio of ~10% and 4 were closer to ~25%. From flow cells with passivation protocols not containing casein, six flow cell sections were taken for tallying, with 10 to 30% stuck bead ratios. The results of our tallying are shown in table S1, where we note how a given bead batch was prepared and the ratio of beads that had stepping versus sliding complexes. Beads that showed solely one, single binding event were not counted as active. These single-event beads were seen when HaloTag ezrin-T567D was incubated with nonfunctionalized BSA-control beads in control experiments and thus may reflect HaloTag ezrin-T567D molecules weakly associated with the passivation layer that are ripped from the bead when subjected to load.

As indicated above, bead batches fall into two categories, those with activity of ≤0.11 (batches I, II, and III) and those with activity of ~0.35 (batches IV, V, VI, and VII). Combining data for batches I, II, and III together and taking weighted averages, we find that 90% were inactive, 7.5% showed stepping complexes, and 2.2% had sliding complexes. For percentages, we calculate an original estimate from long scans as 90, 7.5, and 2.5% in the same order as above; however, sliding complex percentage (2.5%) could be made more precise by incorporating data obtained from short-duration scans, which, as noted above, were designed to detect sliding complexes, but not stepwise detachments. Of the beads with stepping complexes, we detected solely single-step unbinding for 70%, and a mixture of single- and double-step unbinding for 30%. From the beads exhibiting sliding complexes, three were minimal and one was nonminimal. Assuming Poisson statistics and a purely monomeric molecule, at an inactivity ratio of 90%, 9% of total beads are expected to contain single molecules, in reasonable accord with the fraction of beads showing solely single-step unbinding behavior.

Combining data from batches IV, V, VI, and VII and taking weighted averages, we find that 66% of beads showed no activity, 27% had stepping complexes, and had 4.5% sliding complexes, where the percentages do not sum to 100% due to the same considerations as above, where sliding complex percentage is made more precise by incorporating data obtained from short-duration scans. For reference, at 66% inactivity ratio, with same assumptions as above for a purely monomeric molecule, 27% of beads would be expected to contain a single molecule and 6% are expected to contain two molecules.

We interpret above results, where sliding complexes are rarer than complexes showing stepwise release, to indicate that sliding complexes include multiple ezrin-T567D molecules and that the minimal sliding complex likely is formed by the association of two ezrin-T567D molecules with F-actin. We infer that the single-step unbinding we observe at limiting dilutions of 90% bead inactivity is due to single ezrin molecules, as ezrin is known to have a single F-actin–binding site. A Bell-Evans slip bond model is sufficient to explain the observed single-molecule lifetime distribution (Fig. 1D), a finding that supports it arising from a single molecular species. The no-load lifetime we infer, 0.13 s, is in reasonable agreement with that of (*13*), which reports a binding lifetime of 0.77 s based on single-molecule AFM experiments. As is typical for single-molecule force spectroscopy experiments, we cannot completely exclude the possibility that the smallest possible binding unit ezrin forms is a multimolecular complex, rather than a monomer, that exhibits single-step unbinding at the time resolution of our measurement.

For the analysis of minimal sliding complexes, we only included minimal sliding complexes for which data were collected for in batches II, III, and IV since these batches were most completely characterized. In total, minimal versus nonminimal sliding complexes could be assigned for 21 of 23 beads, where we simply did not collect enough information for two beads to confidently ascribe their status as minimal or nonminimal. The partial step unbinding and rebinding events seen in some nonminimal sliding complexes are shown in figs. S3 and S4. Data from batches II and III (~90% inactivity) were also used to calculate the force-dependent lifetime of single molecules when loaded in parallel to the actin filament. Here, we analyzed data collected from beads that solely produced single-step unbinding events (Fig. 1).

#### Bursts and steps exhibited by minimal sliding complexes

As described in the manuscript, in relaxation traces from step loading of minimal sliding complexes, sometimes, bursts (stalls interspersed with sliding) can be seen, which are also apparent in pairwise distance distribution analysis of the some of the traces (figs. S5 and S6). While we do observe bursts at both low (~2 pN) and high (~4 pN) forces, we expect our burst size estimates and temporal resolution to be worse for lower forces due to the higher effective compliance.

#### Slip bond model fitting

The Bell-Evans model for a slip bond (*71*) predicts an exponential dependence of the unbinding rate constant *r* on the applied force *F* as follows

$$r(F)=r(0)\;e^{\frac{F\;d}{k\;T}}$$

where *T* is temperature, *k* is Boltzmann constant, and *d* is the distance parameter. This results in the following exponential probability distribution *P*(τ) for bond lifetime τ

$$P(\tau )=r(0)e^{\frac{F\;d}{k\;T}-r(0)\;\tau \;e^{\frac{F\;d}{k\;T}}}$$

The expression was fit to the minimal sliding complex binding lifetimes under orthogonal force as described in the Fig. 3C legend or to the single-step binding lifetimes as described in Fig. 1D legend. The 2.5 to 97.5% confidence intervals were generated through resampling: The dataset was randomly resampled by the dataset size 1000 times, with each resampling fitted to the slip bond model, and the resulting 2.5 and 97.5 percentile lifetimes at each force value were taken. Assuming an equilibrium dissociation constant of ~5 μM measured in (*33*), our estimate for the mean lifetime at zero force for single ezrin-T567D binding to F-actin (0.13 s; Fig. 1D) suggests an on-rate of ~2 × 10^6^ M^−1^ s^−1^ for the ezrin and F-actin interaction.

## Notes

View/request a protocol for this paper from *Bio-protocol*.
