# Supplementary material for: The membrane-actin linker ezrin acts as a sliding anchor
Source: Sci Adv. 2022 Aug 5;8(31):eabo2779. doi: 10.1126/sciadv.abo2779 (PMC9355349; doi:10.1126/sciadv.abo2779)
Supplement: Supplementary file 1 — Figs. S1 to S7 Table S1 [file sciadv.abo2779_sm.pdf]

Science Advances
AAAS

Supplementary Materials for

The membrane-actin linker ezrin acts as a sliding anchor

Elgin Korkmazhan and Alexander R. Dunn

Corresponding author: Alexander R. Dunn, alex.dunn@stanford.edu

Sci. Adv. **8**, eabo2779 (2022)
DOI: 10.1126/sciadv.abo2779

The PDF file includes:

Figs. S1 to S7
Table S1
Legend for data file S1

Other Supplementary Material for this manuscript includes the following:

Data file S1

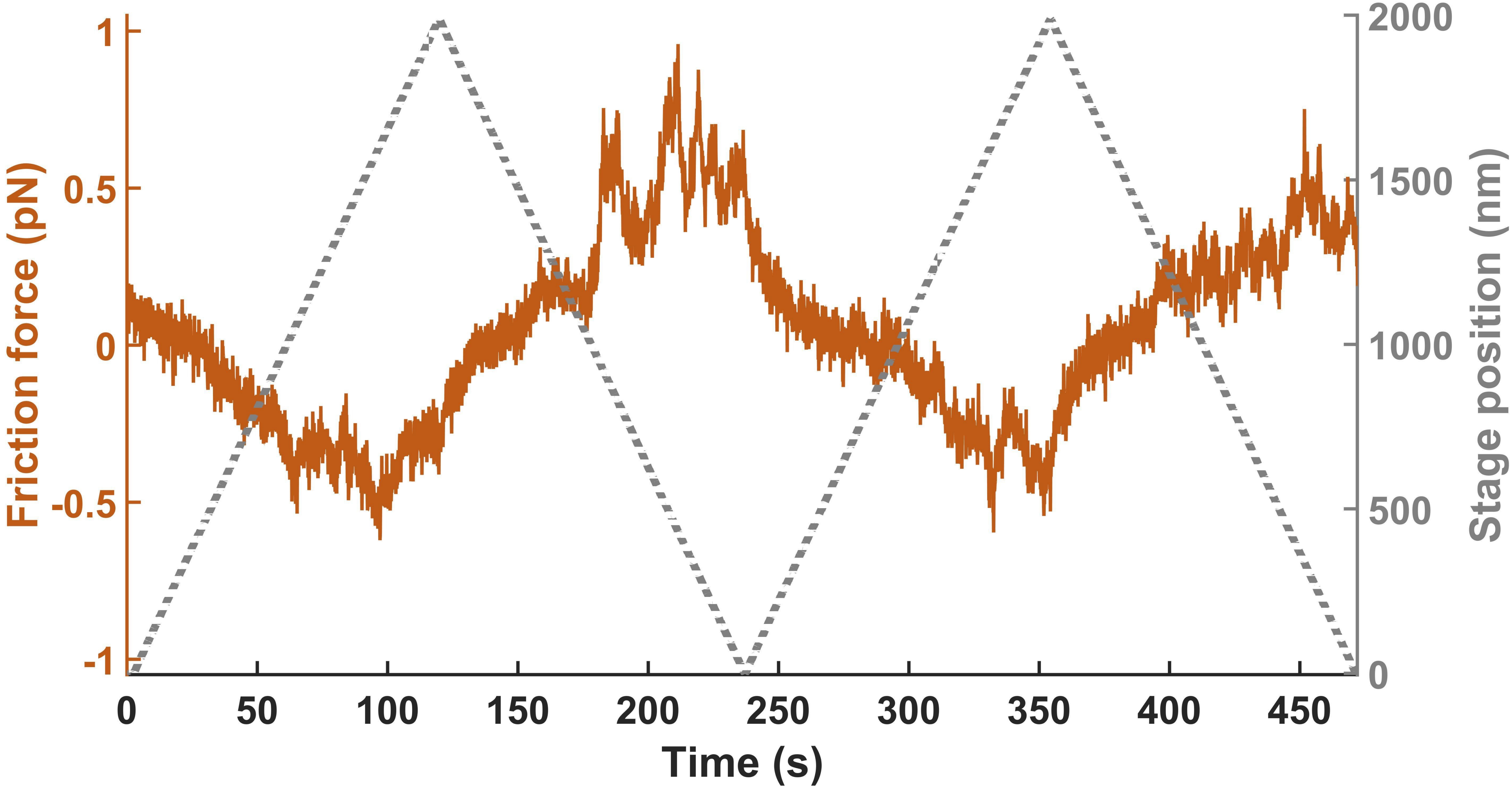

Fig. S1. A minimal sliding complex of ezrin-T567D sliding on F-actin for four consecutive stage ramps. Here, the stage is translated in one direction along the actin filament at 17 nm/s for 2000 nm before reversing its direction to start a new ramp. The minimal sliding complex exerts a friction force in the direction opposite to the stage movement. Force trace is boxcar averaged to 10 Hz.

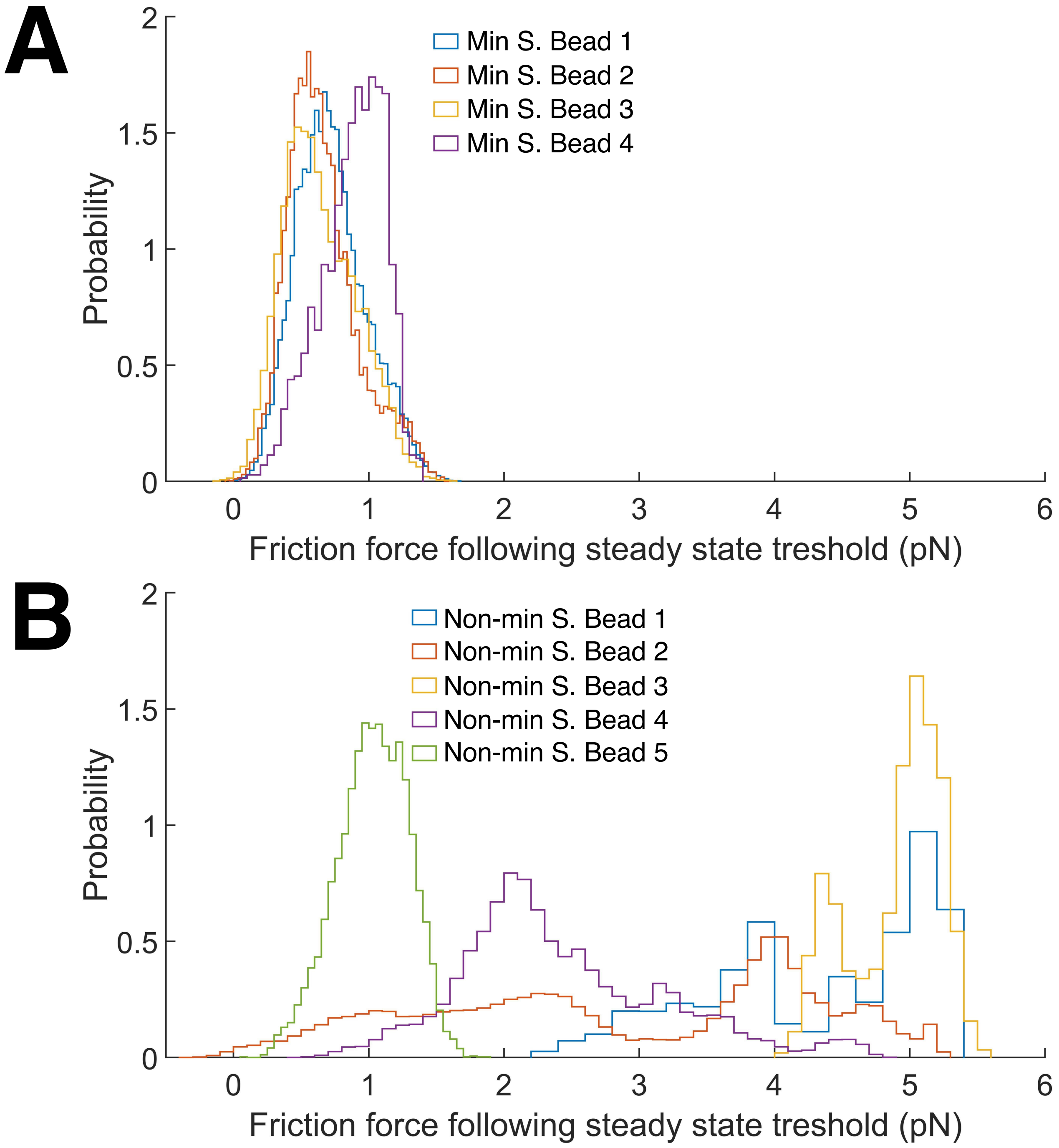

Fig. S2. Friction force distributions following steady-state threshold. (A) The average steady-state friction force distributions for minimal sliding (Min S.) complexes at 34 nm/s after applying steady-state analysis to beads from batch II (Min S. Bead 1), batch III (Min S. Beads 3, 4) and batch IV (Min S. Bead 2) (Table S1; Methods). **(B)** Non-minimal sliding (Non-min S.) complexes at 34 nm/s exhibit markedly different friction force distributions when the same steady-state analysis is applied (Non-min S. Beads 1, 2, 3, 4 from batch IV; Non-min S. Bead 5 from batch III) due to heterogeneity in their steady-state friction forces (Fig. S3), and due to taking longer to approach the larger steady-state forces (Fig. 4b; Fig. S3, S4). While one bead with a non-minimal sliding complex (Non-min S. Bead 5) exhibits a friction force distribution similar to that of minimal sliding complexes at steady-state, it was assigned as non-minimal due to exhibiting multiple cases of partial step unbinding when approaching high forces, which is a phenomenon seen in other non-minimal sliding complexes (Fig. S4) but not in minimal sliding complexes in constant stage ramp experiments.

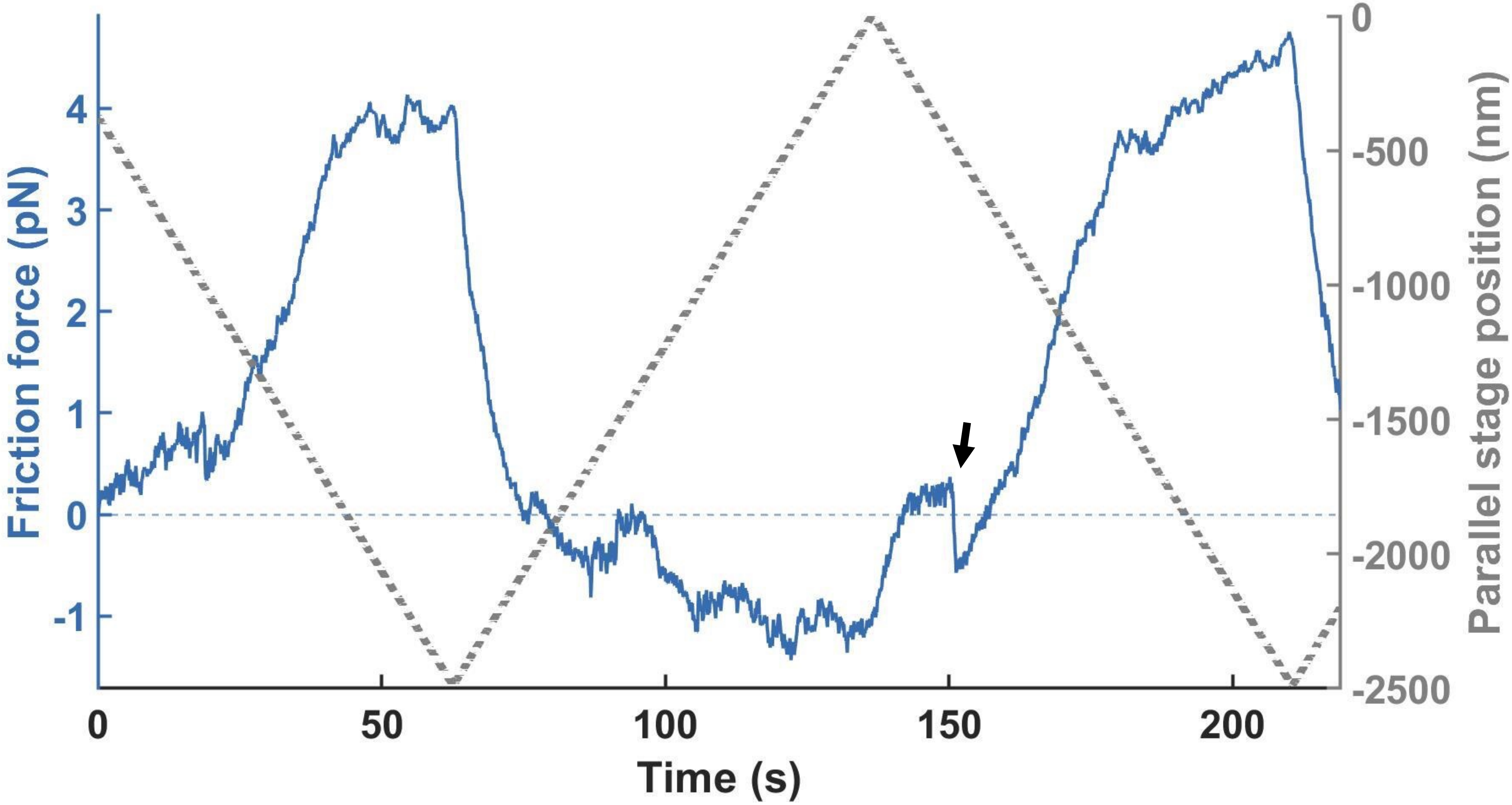

Fig. S3. Non-minimal sliding complexes can exhibit larger and heterogeneous friction forces compared to minimal sliding complexes. A non-minimal sliding complex is seen to approach different steady-state friction forces when the stage is ramped at 34 nm/s in opposite directions along the filament. This may reflect different numbers of ezrin-T567D molecules bound to F-actin when the bead leans in a given direction. The force step indicated by the arrow at ~150 s can be interpreted as the rebinding of an additional complex. This would be consistent with the higher friction force reached in the rest of the ramp (~4 pN at ~190 s compared to ~1 pN at ~130 s in the opposite direction). Force trace is boxcar averaged to 10 Hz.

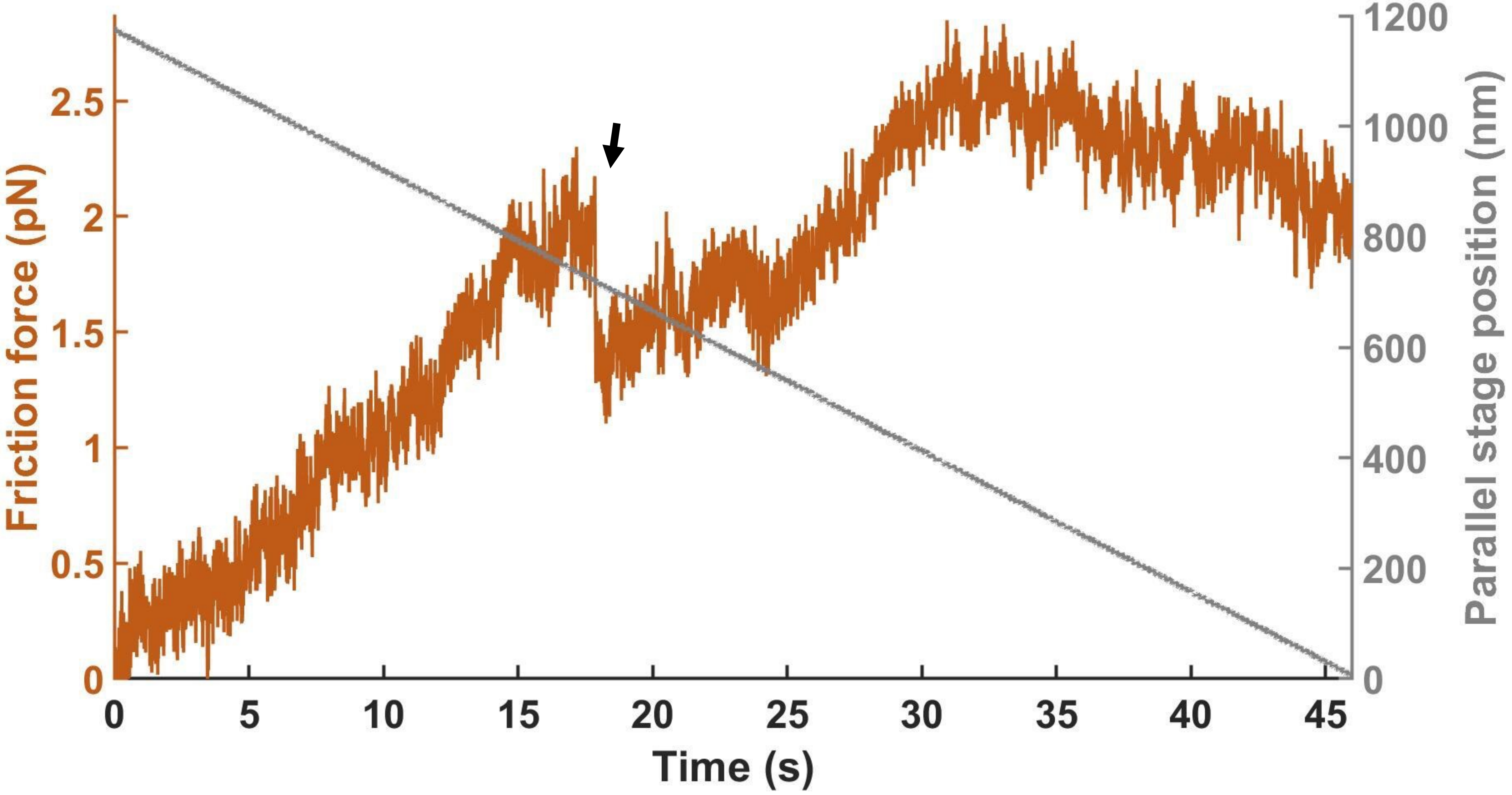

Fig. S4. Friction force of a non-minimal sliding complex at a constant stage velocity of 17 nm/s. An example partial unbinding event of magnitude ~0.5 pN at ~17 s during sliding is indicated with an arrow. Force trace is boxcar averaged to 100 Hz.

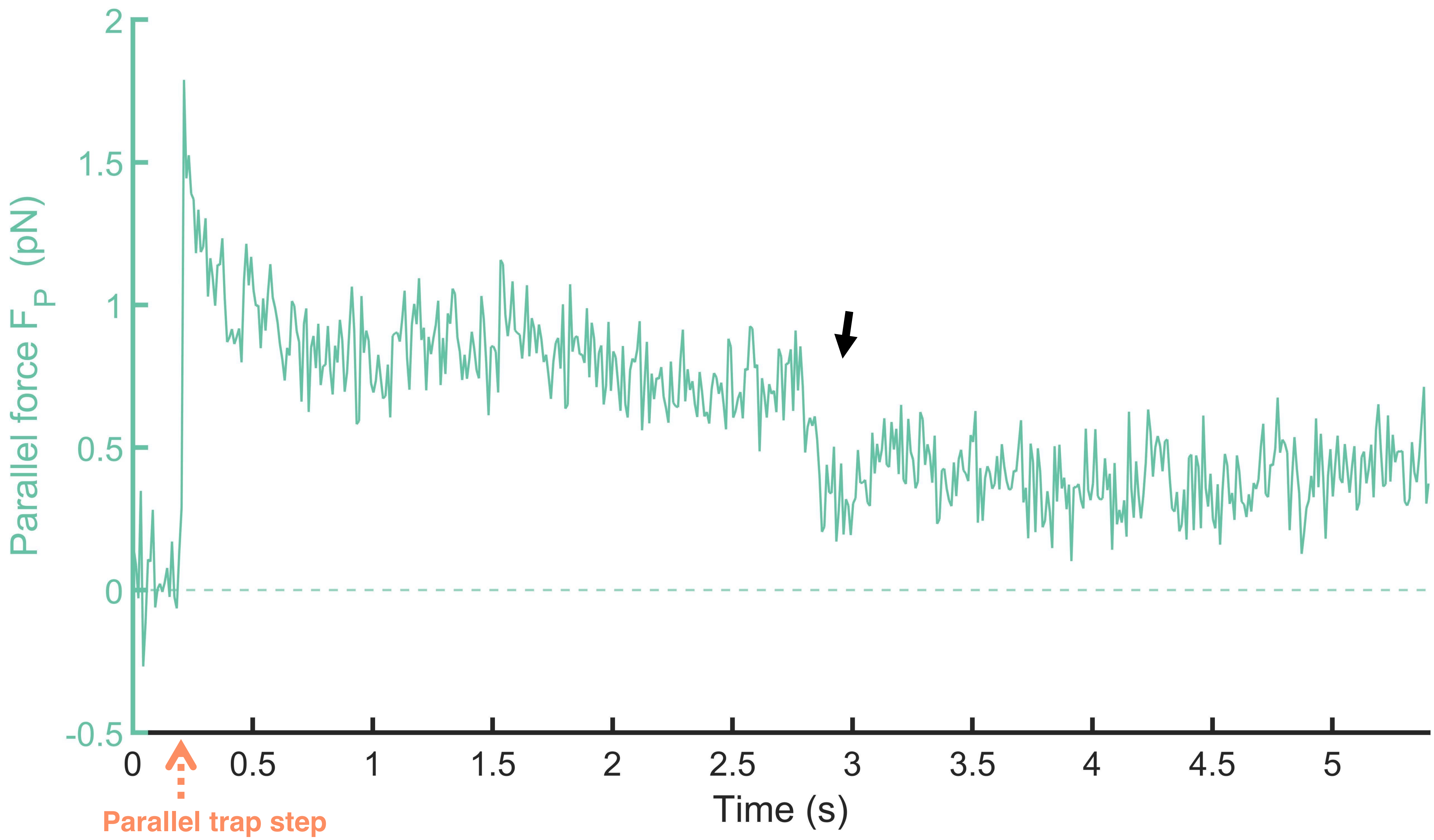

Fig. S5. Minimal sliding complexes can exhibit >10 nm bursts during sliding. A step perturbation parallel to the filament is applied by a step movement of the trap along the filament at ~0.2 s (orange arrow; trap position is not shown) increasing the parallel force to ~1.5 pN, after which the force relaxes though complex sliding. An example ~20 nm burst (arrow). Force trace is boxcar averaged to 100 Hz. Compare to Fig. 2A and Fig. S6.

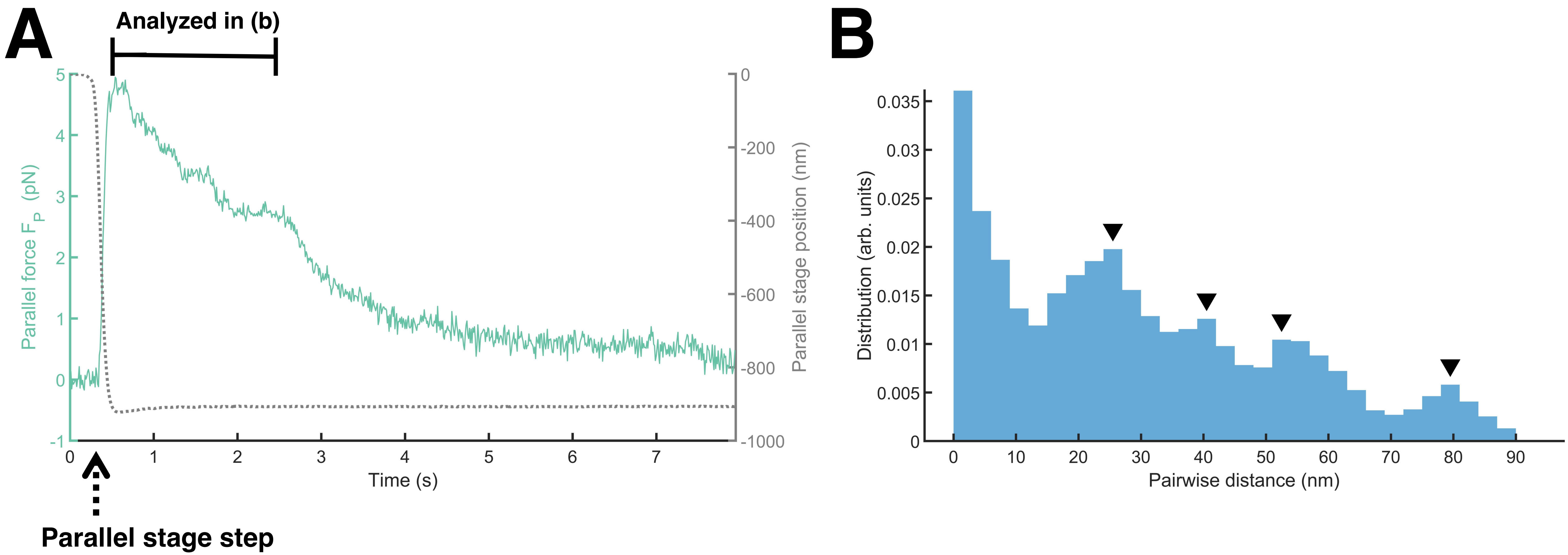

Fig. S6. Pairwise distance analysis of >10 nm bursts of a minimal sliding complex during sliding. (A) A step perturbation raises the parallel force to ~5 pN, after which the force on a minimal sliding complex relaxes to close to zero. **(B)** The pairwise distance distribution corresponding to ~0.5 to 2.6 s in (A). Estimates of burst sizes are indicated by the first peak at 25.5 nm and the following inter-peak distances of 15 nm, 12 nm, 27 nm, as indicated by triangles.

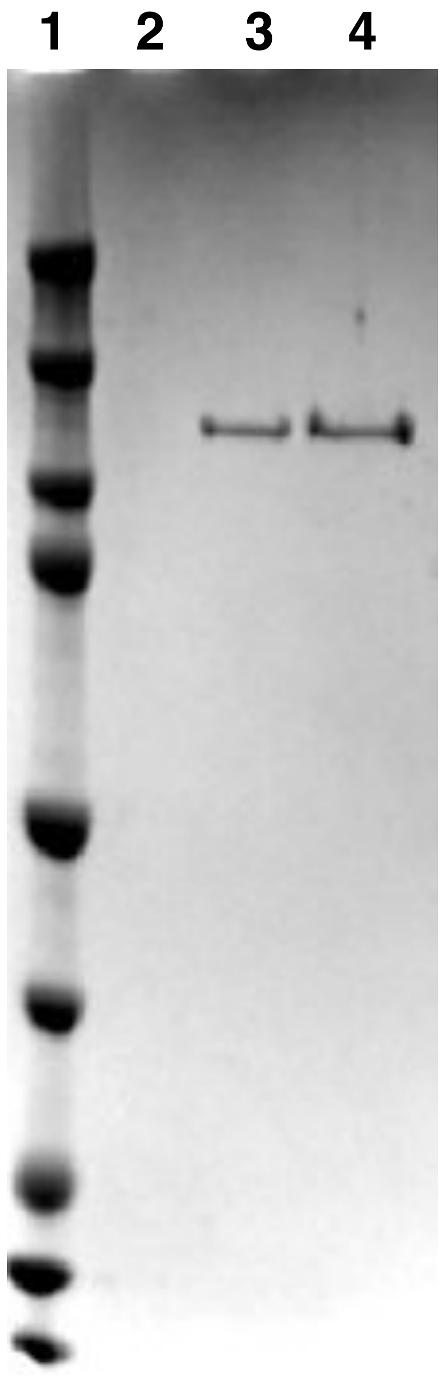

Fig. S7. SDS-PAGE gel of the purified ezrin construct. Lane 1 contains BIO-RAD Dual Color Precision Plus Protein™ Standard. Lane 2 is empty. Lanes 3 and 4 were loaded with different amounts of the purified ezrin construct used in experiments, 6xHis-HaloTag-ezrinT567D, heated in sample loading buffer.

HaloTag fusion ezrin-T567D concentration at incubation (nM)	Bead concentration during incubation with ezrin-T567D (mg/ml)	Incubation time (min)	Batch ID	Long Scan Active bead fraction	Long Scan Stepwise detaching bead fraction	Long Scan Sliding bead fraction	Long Scan Total assayed beads	Short Scan Sliding bead fraction	Short Scan Total assayed beads
12	2.04	2	**I**	0.06	0.06	0.00	16		
49	0.48	40	**II**	0.10	0.10	0.00	42		
26	0.5	75	**III**	0.11	0.06	0.05	62	0.02	65
23	0.5	37	**IV**	0.35	0.29	0.05	75	0.03	267
131	0.41	32	**V**	0.30	0.10	0.20	10		
129	0.56	36	**VI**	0.32	0.25	0.07	28		
4	1.1	15	**VII**	0.38	0.29	0.08	24		

Table S1. Preparation details and bead batch activity statistics. Labeling conditions for different batches of optical trap beads. All BSA-Halo-ligand beads were functionalized with Halo-ligand with the same protocol except for Batch VII where a different protocol was used (Methods). For each bead batch, we collected bead activity statistics by scanning multiple beads to estimate the fraction of active beads, as well as the fraction of beads exhibiting stepwise detachment or sliding. A Long Scanning protocol (LS) determined whether and how a bead was active (i.e. stepwise detachment or sliding) while a short scanning protocol only determined whether a bead exhibited sliding or not (Methods).

Data File S1. Collection of analyzed data. Each sheet contains data points for the indicated figure panel (Figs. 1d, 3a, 3b, 3c), as labeled in the sheet name and within column 1.
